# Performance effects of periodized carbohydrate restriction in endurance trained athletes – a systematic review and meta-analysis

**DOI:** 10.1186/s12970-021-00435-3

**Published:** 2021-05-17

**Authors:** Kasper Degn Gejl, Lars Nybo

**Affiliations:** 1 Department of Sports Science and Clinical Biomechanics, University of Southern Denmark, Campusvej 55, 5230 Odense, Denmark; 2 Department of Nutrition, Exercise and Sports, University of Copenhagen, Copenhagen, Denmark

**Keywords:** Train-low, Carbohydrate periodization, Diet manipulation, Endurance performance, Glycogen, Elite athletes, Endurance sport, Cycling, Triathlon, Race walking

## Abstract

Endurance athletes typically consume carbohydrate-rich diets to allow for optimal performance during competitions and intense training. However, acute exercise studies have revealed that training or recovery with low muscle glycogen stimulates factors of importance for mitochondrial biogenesis in addition to favourable metabolic adaptations in trained athletes. Compromised training quality and particularly lower intensities in peak intervals seem to be a major drawback from dietary interventions with chronic carbohydrate (CHO) restriction. Therefore, the concept of undertaking only selected training sessions with restricted CHO availability (periodized CHO restriction) has been proposed for endurance athletes. However, the overall performance effect of this concept has not been systematically reviewed in highly adapted endurance-trained athletes. We therefore conducted a meta-analysis of training studies that fulfilled the following criteria: *a)* inclusion of females and males demonstrating a VO_2_max ≥ 55 and 60 ml · kg^− 1^ · min^− 1^, respectively; *b)* total intervention and training periods ≥ 1 week, *c)* use of interventions including training and/or recovery with periodized carbohydrate restriction at least three times per week, and *d)* measurements of endurance performance before and after the training period. The literature search resulted in 407 papers of which nine studies fulfilled the inclusion criteria. The subsequent meta-analysis demonstrated no overall effect of CHO periodization on endurance performance compared to control endurance training with normal (high) CHO availability (standardized mean difference = 0.17 [− 0.15, 0.49]; *P* = 0.29). Based on the available literature, we therefore conclude that periodized CHO restriction does not per se enhance performance in endurance-trained athletes. The review discusses different approaches to CHO periodization across studies with a focus on identifying potential physiological benefits.

## Background

The interaction between training and dietary interventions has a long research tradition and for almost a century, it has been recognized that consuming a high-carbohydrate (CHO) diet can enhance endurance performance [1], whereas consumption of a fat-rich diet reduces time to exhaustion, although it increases fat oxidation at a given sub-maximal exercise intensity. Introduction of the muscle biopsy technique in the 1960s by Bergstrøm and colleagues [2] revealed that these findings were linked to muscle glycogen availability and several studies have subsequently confirmed that commencing exercise with low muscle glycogen availability can markedly compromise endurance performance. Accordingly, sports nutrition guidelines recommend that endurance athletes maximize CHO availability during endurance sport competitions, high-intensity training sessions and periods including high overall training loads [3].

However, the conception of the role of CHO for training and competition has become more varied during the past decades. In the early 1980s, Phinney exposed a group of trained cyclists to a chronic ketogenic low CHO high-fat (LCHF) diet (80% of energy from fat) for 4 weeks [4], and observed a significant shift towards a higher reliance on fat during exercise in the fasted state, without a compromised endurance capacity. This increase in the capacity for fat oxidation has since been established in several studies including LCHF diets both with and without ketosis [5]. Due to the large endogenous availability of fat, such manipulations of the macronutrient intake were proposed as a strategy to spare muscle glycogen and potentially improve endurance performance. However, and as recently reviewed by Burke [5], prolonged periods with chronic CHO restriction (i.e., LCHF diets) does not lead to performance enhancements and in some studies and cases adherence to LCHF diets have been shown to be detrimental to performance under “real-life” training conditions of world-class athletes [6–8]. More specifically, a lack of performance enhancement has been observed in intervention studies exposing athletes to chronic LCHF diets for up to 4 weeks [6, 8–12], as well as studies with a short-term adaptation to LCHF (i.e., 5–10 days) before returning to a high-CHO diet during the final lead-in to a performance test [7, 11, 13–16]. In general, LCHF diets have been associated with an impaired ability to perform high intensity exercise, a reduced CHO oxidative capacity, a lower energy yield per litre of O_2_, and reduced mitochondrial respiration, and together this can explain the absent effects of this diet on performance in elite endurance athletes [17, 18].

While adherence to a LCHF diet aims to change the substrate utilization and increase the reliance on fat during exercise, a different nutritional approach including periodic CHO restriction has emerged as a promising way to amplify the acute response to endurance training [19]. It is well-established that endurance training leads to the creation of new mitochondrial reticular components (i.e., mitochondrial biogenesis) [20]. Accordingly, the skeletal muscles of elite endurance athletes are characterized by a higher mitochondrial volume, cristae density and function in comparison to less trained individuals [21, 22]. Altogether, these alterations ultimately increase the aerobic performance capacity of the skeletal muscles and consequently, a primary goal of endurance training is to maximize the mitochondrial biogenesis. In this regard, commencing and completing prolonged exercise bouts with reduced CHO availability has been suggested as a way to amplify the mitochondrial biogenesis. For instance, pioneering studies by Pilegaard and colleagues [23, 24] demonstrated that cell signalling pathways that promote a reinforcement of the skeletal muscle metabolism (i.e., mitochondrial biogenesis) were acutely enhanced when endurance exercise was commenced with low muscle glycogen availability (“train-low”). These observations were later supported by findings in studies of both recreationally active individuals [25–27] and endurance athletes [28–31]. Specifically, the training induced activation of AMP-activated protein kinase (AMPK), a key regulator of cellular energy homeostasis, seems to be enhanced when the CHO availability is reduced [32]. Since AMPK activation has been shown to induce mitochondrial biogenesis through its activation of the transcription factor p53 and peroxisome proliferator-activated receptor γ co-activator-1 α (PGC-1α) and downstream targets in the mitochondria (e.g., Tfam, COX subunits, MFN-2, DRP-1), a reduced CHO availability during exercise may advance the development of the mitochondrial network [33–35].

To benefit from the potential attractive adaptations related to training with low CHO availability, training interventions including CHO restriction around selected sessions have been studied in both trained athletes and untrained individuals [32]. Since the pioneering study by Hansen et al. from 2004 [36], several different approaches to manipulate the CHO availability during training and recovery have been presented [32], which has led to both confusion and miscommunication in the elite sport community about the variety of terms related to CHO manipulation. Accordingly, this recently gave rise the proposal of a set of definitions by Burke and colleagues, attempting to create a common understanding of diet-exercise strategies [19]. Despite their different nuances, studies in the existing literature can be divided into the following: *1)* “Twice-a-day training” that involves a training session designed to deplete muscle glycogen, followed by recovery with CHO restriction or fasting and a second training session commenced with low muscle glycogen levels, *2)* “Sleep low” which refers to a glycogen-depleting session of training followed by overnight CHO restriction or fasting followed by a training session in the morning, *3)* “Fasted training” by conduction of endurance training without CHO provision or *4)* “Recover low” with a single glycogen-depleting training session followed by recovery with CHO restriction or fasting.

While studies of the acute effects of these CHO periodization models have mainly investigated changes in cell signalling and transcriptional responses promoting fat metabolism and mitochondrial biogenesis, training studies have evaluated the translation of these responses into persistent adaptations with importance to performance. To be able to provide specific recommendations about CHO periodization for athletes, it is important to stratify by training status since trained endurance athletes are highly metabolically adapted and less susceptible to exercise-induced stress when compared to that of untrained individuals [37, 38]. Therefore, in this systematic review, we present an overview and meta-analysis of studies investigating performance effects of periodizing the CHO availability in well-trained endurance athletes.

## Methods

### Literature search

**Fig. 1:**
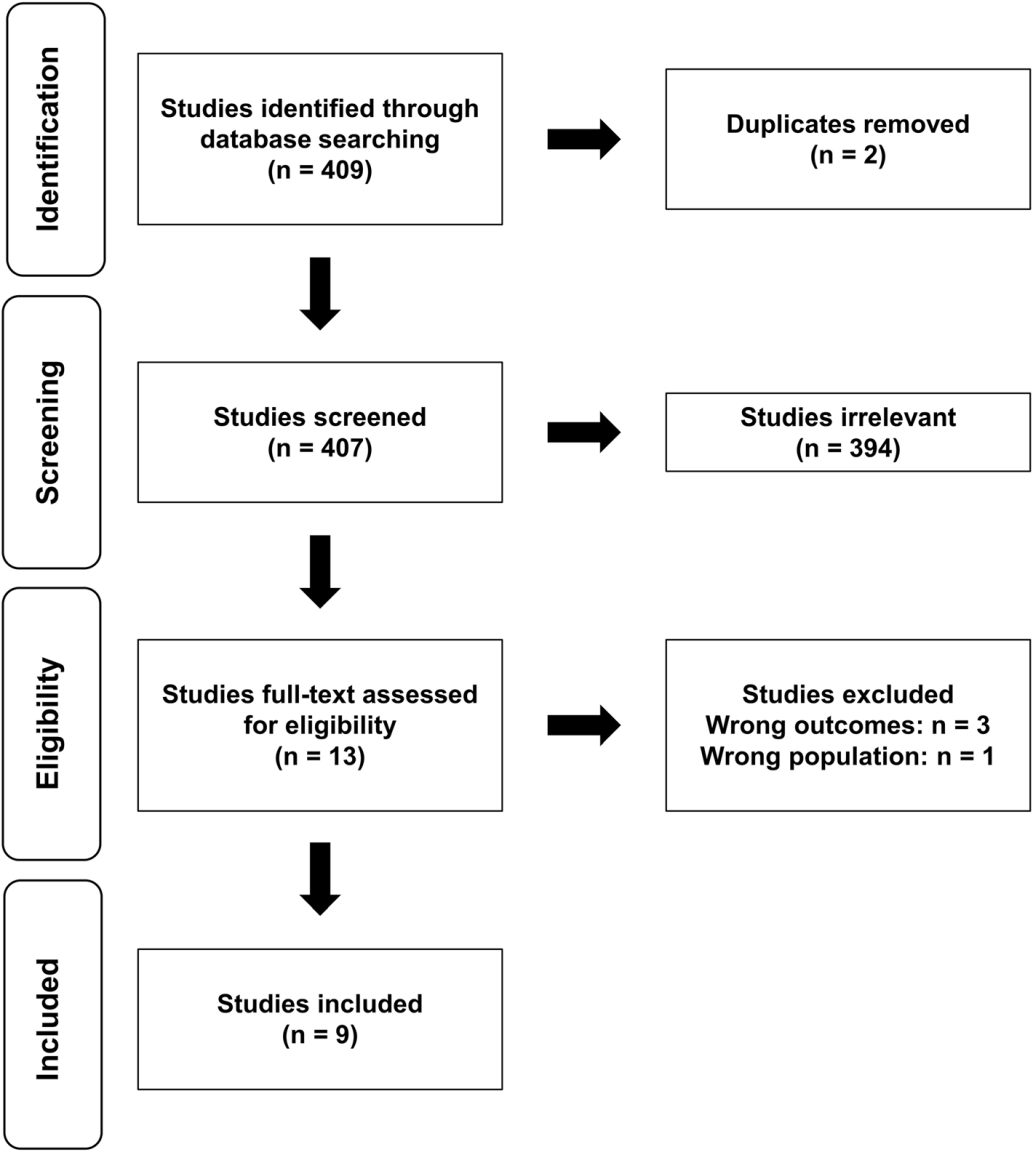
Flow diagram showing the different steps of the systematic review, starting from the literature search to study selection and exclusion

This present paper and associated meta-analysis are based on a systematic search and screening strategy to identify all relevant publications (see Fig. 1 for overview). Relevant studies were identified by a literature search in the online databases Pubmed (Medline) and SPORTDiscus in October 2020. The primary search syntax included elements about populations, interventions and outcomes relevant to the purpose of the present review, and was constructed as follows: ((elite) OR (athlete*) OR (trained) OR (triathlete*) OR (cyclist*) OR (runner*)) AND ((“train low”) OR (“train-low”) OR (“sleep low”) OR (“sleep-low”) OR (“periodized nutrition”) OR (“carbohydrate availability”) OR (“CHO availability”) OR (“carbohydrate periodization”) OR (“CHO periodization”) OR (“carbohydrate manipulation”) OR (“CHO manipulation”) OR (“glycogen availability”) OR (“glycogen manipulation”) OR (“low muscle glycogen”) OR (“glycogen depletion”)) AND ((performance) OR (“time to exhaustion”) OR (“time trial*”)). From the search, all titles and abstracts were initially screened by both authors according to the following inclusion criteria: *a)* female and male athletes demonstrating a VO_2_max ≥ 55 ml · kg^− 1^ · min^− 1^ and VO_2_max ≥ 60 ml · kg^− 1^ · min^− 1^, respectively; *b)* training periods lasting ≥ 1 week, *c)* use of interventions including training and/or recovery with periodized CHO restriction at least three times per week, and d*)* a determination of the effects of CHO periodization on endurance performance. Studies were excluded if the intervention involved long periods (i.e., days) with a chronic LCHF diet (i.e., no CHO periodization). If studies were considered relevant or if relevance could not be determined from the title or abstract, full text articles were reviewed. A secondary search was performed by screening the reference lists of all selected studies. Reviews and case studies were excluded.

### Methodological quality assessment

The Physiotherapy Evidence Database (PEDro) scale was used to assess the quality of the included studies [39] (Table 1). The PEDro scale includes an 11-point scoring system assessing: *1)* specified eligibility criteria, *2)* random treatment allocation, *3)* concealed treatment allocation, *4)* similarity between groups at baseline, *5)* subject blinding, *6)* therapist blinding, *7)* assessor blinding, *8)* completeness of follow-up, *9)* conduction of intention-to-treat analysis, *10)* results of between-group statistical comparisons of key outcomes (i.e., endurance performance), and *11)* point measures and variability. The PEDro scale rates studies from 0 to 10 points, with one point potentially being awarded for each item. Item #1, used to assess the eligibility criteria, is not included in the final score. Points were only awarded when a criterion was clearly satisfied. So, to receive one point, it had to be explicitly stated in the manuscript that the criterion was met. One person performed the quality assessment and in case of uncertainty a second person was invited to assist the quality assessment.

**Table 1:** Overview of the subjects, strategies, performance changes and PEDro scores of the included studies

Study	Subjects	VO_**2**_max (ml·kg^**− 1**^·min^**− 1**^)	”Train-low” strategy	Changes in endurance performance	PEDro score
Yeo et al. 2008 [40]	14 male cyclists Low: *n* = 7 High: n = 7	Low: 60 High: 61	”Twice-a-day” every second day vs. “Once-a-day” every day 6 sessions (3 AT and 3 HIIT) per week for 3 weeks HIIT commenced with low vs. high muscle glycogen Athletes instructed to consume of 8-9 g CHO ·kg^− 1^·day^− 1^ throughout the period Total training volume: 7 h·week^− 1^	Low = High 60 min preload + 60 min TT 10–12% increase in PO during TT in both groups (*P* < 0.01)	5
Hulston et al. 2010 [41]	14 male cyclists Low: n = 7 High: n = 7	Low: 65 High: 66	”Twice-a-day” every second day vs. “Once-a-day” every day 6 sessions (3 AT and 3 HIIT) per week for 3 weeks HIIT commenced with low vs. high CHO availability Athletes instructed to consume high CHO diet throughout the period Total training volume: 7.5 h·week^− 1^	Low = High 60 min preload + 1017 ± 73 kJ TT 10–11% increase in PO during TT in both groups (*P* < 0.001)	6
Cox et al. 2010 [42]	16 male triathletes Low: *n* = 8 High: n = 8	Groups combined: 65	“Fasted training” vs. training with high CHO availability 6 sessions per week for 23 days 5 g CHO·kg^− 1^·day^− 1^ in both groups High: + 1.5 g CHO·kg^− 1^·day^− 1^ for every hour of exercise Low: + 25 kJ·kg^− 1^·day^− 1^ for every hour of exercise from fat and protein Low: fasting for 2 h prior to and during all sessions High: ingestion of CHO before or during all sessions	Low = High 100 min preload + 7 kJ/kg TT 4–6% reduced time in TT in both groups (*P* < 0.01)	6
Marquet et al. 2016 [43]	21 male triathletes Low: *n* = 11 High: *n* = 10	Low: 60 High: 60	”Sleep-low” 3x CHO periodization per week for 3 weeks Identical diets in both groups but different timing 0 g vs. 5 g CHO·kg^− 1^ during and between afternoon and morning sessions Additional AT sessions in both groups Total training volume: 10-15 h·week^− 1^	Low > High 40 km bike preload + 10 km run − 2.9% run time in Low (*P* < 0.01) − 0.1% run time in High (ns)	6
Marquet et al. 2016 [44]	21 male cyclists Low: *n* = 12 High: *n* = 9	Groups combined: 64	”Sleep-low” 3x per week for 1 week Identical diets in Low and High around “train-low” sessions but different timing 0 vs. 5 g CHO·kg^− 1^ between afternoon HIIT and morning AT. Total training volume: 5 h during the 6-day training period.	Low > High 120 min preload + 20 km TT − 3% TT time in Low (*P* < 0.05) − 1% TT time in High (ns)	6
Burke et al. 2017 [6]	19 male elite race walkers Low: n = 10 High: n = 9	Low: 65 High: 62	“Fasted training”, “Sleep-low” and “Twice-a-day” Alternating strategies 6 days per week for 3 weeks Identical energy and CHO intake low and high but different timing. Average total training volume: Low: 125 km·week^− 1^; High: 117 km·week^− 1^	Low = High 10 km race walking 5–7% reduced walking time in both groups (*P* < 0.01)	5
Gejl et al. 2017 [45]	26 male triathletes and cyclists Low: *n* = 13 High: n = 13	Low: 65 High: 65	”Twice-a-day” 6 sessions (3 HIT and 3 AT) per week for 4 weeks 1 g vs. 6 g CHO · kg^− 1^ between HIT and LIT sessions (isocaloric diets) Additional AT sessions to attain habitual training volume Average total training volume in matched pairs of athletes: 16 h·week^− 1^	Low = High 90 min preload + 30 min TT 5–6% increase in PO during TT in both groups (*P* = 0.0003)	6
Riis et al. 2019 [46]	13 male endurance athletes Low: *n* = 6 High: n = 7	Low: 63 High: 65	”Sleep-low” 6 sessions (3 HIT and 3 AT) per week for 4 weeks Identical diets but different timing. 0 vs. 3.6 g CHO·kg^− 1^ between afternoon HIIT and morning AT Total training volume: 6hrs45min·week^− 1^	Low = High 90 min preload + 30 min TT 14–19% increase in PO during TT in both groups (*P* = 0.005)	7
Burke et al. 2020 [8]	16 elite race walkers Low: *n* = 5 males *n* = 3 females High: n = 5 males n = 3 females	Low: 59 High: 58	“Fasted training”, “Sleep-low” and “Twice-a-day” Alternating strategies 6 days per week for 25 days Identical energy intake in Low and High but different timing Average total training volume: 113 and 106 km·week^− 1^ in Low and High.	Low = High 10 km race walk − 2.2% walk time in Low (*P* = 0.09) − 4.8% walk time in High (*P* < 0.001)	5

AT moderate-to-high intensity aerobic training, CHO carbohydrate, High control group receiving CHO, HIIT high intensity interval training, LIT low intensity training session, Low “train-low” group exposed to periodized fasting or CHO restriction, MAP maximal aerobic power, PO power output, TT cycling time trial, VO_2_max maximal oxygen consumption

### Data analysis

To calculate effect sizes, individual measures of performance before and after interventions were obtained either directly from the manuscripts, by contacting authors or manually by reading figures using appropriate software (WebPlotDigitizer). This software was calibrated according to the axes of the figures to ensure a precise reading of data. The meta-analysis was conducted including one measure of endurance performance from each of the included studies. Measures of endurance performance varied between the included studies; five studies used power output assessed during a cycling time-trial [40, 41, 44–46], while three studies evaluated endurance performance as the time to complete either 10 km of running following 40 km of cycling in triathletes [43] or 10 km of race walking [6, 8]. In studies determining endurance performance in both fasted and CHO-fed states, results from the performance test conducted in the CHO-fed state were included. To describe the observed intervention effects, standardized mean differences (SMD) were calculated based on mean relative change from baseline to post for the periodized and high CHO groups in the included studies. Positive SMDs represent an effect in favour of the intervention group (i.e., train-low / periodized CHO), while negative SMDs represent an effect in favour of the control groups (i.e., high-CHO). Afterwards, a combined intervention effect estimate was calculated as a weighted average of the estimated SMDs. Due to the homogeneity of the included studies in combination with a relatively low number of studies with small sample sizes, a fixed-effect model was applied. In accordance with the Cochrane recommendation [47], heterogeneity across studies and its impact on the meta-analysis were assessed using chi^2^ and I^2^, respectively. Based on I^2^, the impact of heterogeneity on the meta-analysis may be considered as following: 0 to 40%: might not be important; 30 to 60%: may represent moderate heterogeneity; 50 to 90%: may represent substantial heterogeneity; 75 to 100%: considerable heterogeneity. Due to the low number of studies, reasons for heterogeneity were not explored. However, in case of indications of heterogeneity (Chi^2^ *P* < 0.1 and I^2^ > 30%), additional analyses were conducted using a random-effect model to account for the variability between studies. The analyses were conducted using Review Manager 5.4 (RevMan Web 2020, The Cochrane Collaboration).

Besides endurance performance, various outcomes were determined in the included studies but without consistency between studies. Therefore, meta-analyses were not conducted for these parameters (e.g., enzyme activity, fat oxidation rate etc.). In this regard, substrate utilization has been measured in the fasted state in some studies and in a fed state in others, thus further complicating a comparison hereof.

## Results

In total, the literature search resulted in 409 citations of which two were duplicates (Fig. 1). Following screening of titles, abstracts and identification of duplicates, 394 papers were excluded since they clearly did not comply with the inclusion criteria, leaving 13 publications for full text review (Fig. 1). The main reasons for exclusion of studies in the initial screening were either related to training status of the participants not meeting the inclusion criteria, or that studies did not investigate effects of CHO periodization on endurance performance. Following the full-text review of the 13 identified papers, an additional four studies were excluded due to similar reasons, as mentioned above. Screening the references of the remaining studies did not result in identification of additional studies meeting the inclusion criteria, leaving a total of nine studies eligible for inclusion in the meta-analysis (Table 1).

The durations of the included training studies varied from one to 4 weeks and the number of participants from 11 to 26 (Table 1). Three studies utilized the “twice-a-day” training approach [40, 41, 45], another three studies the “sleep-low” approach [43, 44, 46], two studies a mixture of different strategies (i.e., twice-a-day training, fasted training, sleep-low) [6, 8], while one study has employed fasted training [42]. Five of the studies included invasive methods for determination of muscular training adaptations (e.g., mitochondrial enzymes) [40–42, 45, 46]. Using the PEDro scale, the methodological quality of the studies was on average rated 5.8 ± 0.9 of 10 (Table 1). Mainly the studies were different with regard to the use of randomization to allocate subjects to intervention and control groups.

**Fig. 2:**
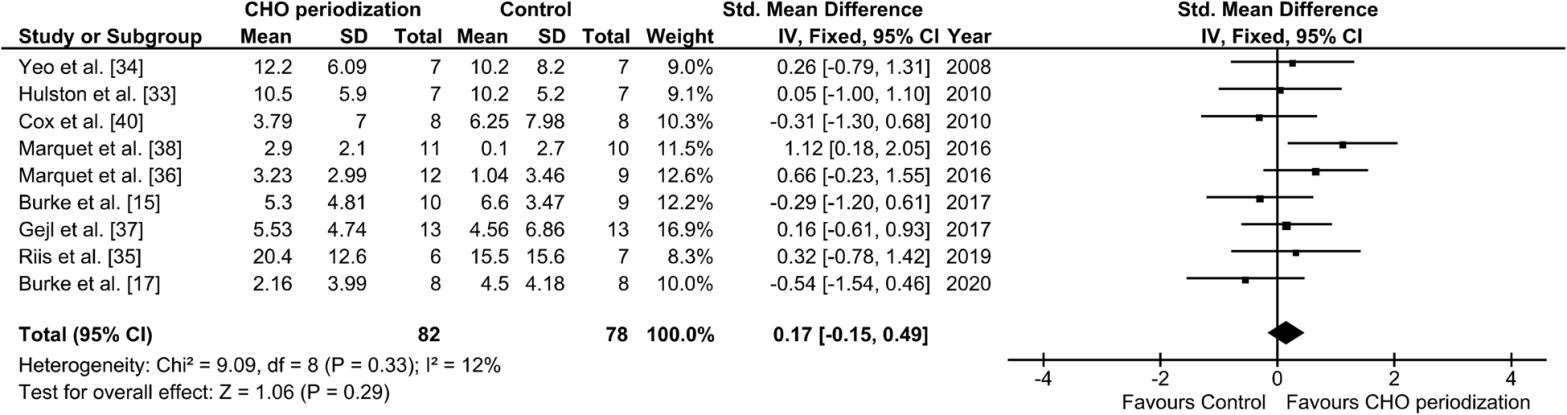
Forest plot illustrating the effects of endurance training with high (Control) vs. periodized CHO intake on endurance performance in well-trained endurance athletes. “Mean” is the mean relative change in endurance performance and “SD” the variation of these relative changes. “Total” is the number of subjects in each group. The x-axis denotes Cohen’s *d* (standardized mean difference). The whiskers indicate 95% confidence intervals

The meta-analysis revealed that the overall effect of periodizing CHO availability on performance in well-trained endurance athletes was not significant, when compared to a chronic high CHO diet (SMD = 0.17 [− 0.15, 0.49]; *P* = 0.29) (Fig. 2 and Table 1). Overall, two of the nine studies showed that performance was improved by training with CHO periodization and not by training in a CHO fed state [43, 44], but no group x time interactions were reported in these studies.

Extraction of muscle tissue was not performed in all studies and in those five studies providing results on the muscular level, different parameters were measured. For that reason, and since the primary outcome in the search strategy was endurance performance, meta-analyses were not conducted for muscular adaptations. In brief, citrate synthase (CS) activity was either enhanced [40], similarly increased [45] or increased to a lesser extent [42] by training with CHO periodization in comparison to training in a CHO fed state. Concerning the β-hydroxyacyl-CoA dehydrogenase (β-HAD) activity, this was increased only by training with CHO periodization in two studies [40, 41], while similar β-HAD activities [42, 45] and -protein contents [46] were observed in CHO fed and -periodized groups in the remaining studies.

## Discussion

Overall, the present meta-analysis does not support periodic CHO restriction as a superior approach for enhancing endurance performance in well-trained athletes. Thus, the physiological stimuli prompted by undertaking an acute exercise bout with low CHO availability (as observed in acute exercise studies [28–31]) does not translate into clear measurable enhancements of performance in already adapted endurance-trained athletes compared to training with high CHO availability (Fig. 2).

This overall meta-analysis was based on the effect on endurance performance in nine studies of well-trained endurance athletes and the evaluation of the methodological quality revealed that these studies achieved five to seven of 10 points on the PEDro scale (Table 1). This scale has previously been interpreted so that studies scoring lower than four points are considered to be of “poor” quality, four to five points “fair” quality, six to eight points “good” quality, while nine to 10 points indicates excellent methodological quality [48]. Overall, the nine studies received an average of 5.8 points, and all the included studies are categorized as either “fair” or “good” quality studies (see Table 1).

In general, nutritional studies like these are difficult to blind, at least to athletes and therapists administering the diets, and accordingly, these two points were not rewarded to any of the studies. Due to this inherent difficulty of blinding nutritional studies, evaluations of the PEDro score can consequently lead to misinterpretations of the scientific merit of these studies. Therefore, such studies must also be interpreted in this context, and with this inherent limitation in mind. The lack of blinding increases the risk of bias, and few studies have circumvented this by allocating the athletes to their preferred treatment [6, 8]. However, this also implies that the criterion of randomization was not met in these studies, which increases the risk of selection bias and bias due to confounding.

Training or recovery with restricted CHO availability may in practice be achieved by numerous combinations of training and dietary interventions but the nine studies identified by the present systematic search can principally be divided into three overall categories. One group of studies [43, 44, 46] employed a “sleep-low strategy” where the CHO intake was restricted between a depleting session in the afternoon and a “train-low” session the subsequent morning. Interestingly, two of the three studies in this category [43, 44] reported superior effects on endurance performance in the groups training with CHO periodization, but as discussed below, this may, at least in part, relate to adaptations not related to “train-low” per se. Another group of studies [40, 41, 45] included two daily training sessions of which the first was intended to deplete muscle glycogen. This session was followed by a CHO restricted recovery period whereby the second session commenced with low CHO availability. All studies in this category reported no superior effect on endurance performance in the CHO restricted groups compared to groups consuming a high CHO diet between the sessions [45] or training once every day with replenished muscle glycogen [40, 41]. The last three studies incorporated CHO periodization by either training with restricted CHO intake or restrictive strategies applied during both training and recovery [6, 8, 42]. All three studies reported no additional improvements of performance in comparison to control groups with a high CHO intake. Although no collective evidence for CHO periodization was shown by the present analysis, it is relevant to nutritionists, coaches and athletes to evaluate the different strategies and identify benefits and side-effects that may affect the overall training outcome.

### Training after overnight recovery with reduced CHO intake

Interestingly the two only studies displaying beneficial overall effects from CHO restriction belong to this category and were conducted by Marquet and colleagues [43, 44] (Table 1). Both studies utilized the sleep-low approach with high-intensity training in the afternoon followed by moderate intensity cycling the subsequent morning (i.e., presumably with low muscle glycogen, although not verified by measures in the two studies). Following 3 weeks of training, 10 km run time, preceded by 40 km preload cycling, was improved by 3% in the sleep-low group [43], whereas it remained unchanged in the control group. Similarly, time to complete a 20 km time-trial was improved by 3% solely in the sleep-low group following an intervention lasting only one week [44]. Unfortunately, potential underlying physiological mechanisms were not investigated in these studies, but as mentioned by the authors, the superior effect after only one week of training may be explained by super-compensated muscle glycogen stores [44]. This notion was supported by numerical increases in the total energy and CHO intake in the CHO periodized group during the one-week training period and the concomitant numerical reductions in the CHO-fed control group. Concerning the study lasting 3 weeks, a significant weight loss was observed in the sleep-low group [43], which may have contributed to the observed change in running performance [49]. Moreover, rate of perceived exertion (RPE) at termination of the 120 min preload cycling was reduced only in the sleep-low group after the training period, suggesting that the improved running performance could also be attributed to a less demanding preload cycling. Endurance performance was not improved in the CHO-fed control groups of these two studies, which could indicate that the training intervention per se was either sub-optimal or that the group of athletes were “performance stable” after the two to three week lead-in periods. It was reported that RPE during the morning sessions was significantly higher in the sleep-low groups of both studies, which indicates that the internal training loads were different between groups and that the control groups could have tolerated a higher training load [43, 44]. If the training load was actually sub-optimal, these findings could indicate that “sleep-low” constitutes a beneficial superimposing strategy during periods with low-to-moderate training loads that per se elicit a submaximal training response. Using a comparable sleep-low approach three days a week for four weeks, Riis and colleagues were not able to demonstrate a superior effect of CHO periodization on endurance performance [46]. In their study, power output during a 30 min time-trial that were preceded by 90 min of preload cycling was improved similarly by 15–19% after receiving high and periodized CHO diets.

### Restriction of CHO intake between two daily sessions

Conduction of two daily sessions can be another way of utilizing a depleting training session to commence both post-exercise recovery and a second training session with reduced CHO availability, although with a shorter recovery period between sessions (i.e., 1-7 h). Yeo et al. [40] and Hulston et al. [41] accomplished comparable studies, including six weekly cycling sessions, alternating between a prolonged session of moderate intensity and a HIIT session. Athletes trained either once every day with high muscle glycogen availability or twice every second day with 1-2 h recovery between sessions. By this approach, the β-HAD activity was solely increased in the groups training twice every second day, which was also the case for CS activity in the study by Yeo et al. [40]. Importantly, training twice per day was associated with a reduced intensity during the HIIT sessions, which may have lowered the training response and outweighed the superior enzymatic adaptations. Thus, endurance performance was not superiorly improved by commencing every second session with low muscle glycogen (Table 1). Importantly, the different training distributions between groups (i.e., once every day vs. twice every second day) leaves a question as to whether the enhanced enzymatic adaptations were due to the periodic CHO restriction or the different training schedules. A recent study in untrained individuals modified the approach by reducing glycogen availability prior to every second session in both groups [50]. Here, it was shown that mitochondrial adaptations were superior in the group training twice per day, indicating that differences in training distributions may have affected the metabolic adaptations, irrespective of differences in muscle glycogen.

We used an alternative approach with two groups training twice per day while consuming isocaloric diets between sessions containing either low or high CHO [45]. As in the above-mentioned sleep-low studies, HIIT was used to reduced muscle glycogen availability, entailing that external training loads were identical in both groups. Three days a week athletes performed high intensity cycling in the morning, recovered for 7 h with a high or low CHO intake and trained for 2 h at a moderate intensity in the afternoon. This intervention was superimposed to the routine training of endurance athletes and following the four-week training period, CS activity and endurance performance were increased to the same extent in both groups. Importantly, a check following the 7th day with CHO manipulation revealed high levels of muscle glycogen after training with the CHO restricted diet (i.e., 431 mmol·kg dw^− 1^). Since low glycogen levels seems to be a prerequisite in order to enhance the acute training response in endurance athletes [28, 29, 31], this could explain the absent superior effects in that study. This observation furthermore demonstrates a need for demanding interventions to repeatedly induce glycogen depletion in endurance athletes. In general, resting glycogen levels increase by endurance training and accordingly we have observed resting levels of up to 880 mmol·kg dw^− 1^ in highly trained endurance athletes [45, 51]. Together with a high metabolic flexibility [40, 46, 52], this obviously counteracts glycogen depletion during exercise. It has been suggested that glycogen levels of 250–300 mmol·kg dw^− 1^ are advantageous in order to provide a cellular environment that facilitates cell signalling [53], and consequently, it seems preferable to commence glycogen depleting sessions with moderate, rather than high, glycogen levels. Otherwise, the glycogen depleting sessions must include a certain amount of moderate-to-high-intensity exercise to reach low glycogen levels, and moreover energy restriction rather than just CHO restriction, may be necessary to maintain a reduced muscle glycogen availability during post-exercise recovery.

### Alternating between different CHO periodization stimuli

Performing the same training protocol repeatedly can lead to a gradual reduction in the acute training response emphasizing a general need for varying the training stimulus [38, 54, 55]. This may also apply to CHO periodization, and to prevent this potential plateau phenomenon, a mixture of different types of training and/or CHO restrictive strategies seems reasonable. In this context, Cox et al. [42] conducted a training study with two groups of cyclists and triathletes completing routine training (e.g., hill rides, HIIT session, prolonged sessions) (Table 1). In one group, CHO was consumed before and during each training session, whereas the other group fasted for 2 h prior to each training session and for the initial 90mins of the extended sessions. Following training, CS activity was increased only in the group training with high CHO availability, while time to complete a 7 kJ·kg bw^− 1^ time trial following 100 min of preload was similarly improved in both groups (Table 1). In contrast, β-HAD activity remained unchanged in both groups. In other studies by Burke and colleagues [6, 8], elite race walkers completed a period of routine intensified training with either high CHO availability or with alternation between different CHO periodizing strategies (i.e., “fasted training”, “twice-a-day” training, and “sleep-low”) (Table 1). Following 3 weeks of training, no superior effect of CHO periodization was observed on 10 km race time and improvements were comparable to those observed following training with high CHO availability.

Overall, seven of the nine studies have revealed that training with CHO periodization is not superior in terms of endurance performance when compared to a high CHO diet in highly adapted athletes. In addition to the above-mentioned possible explanations of these similarities, it cannot be excluded that the conduction of glycogen depleting sessions in the CHO-fed control groups did initiate cell signalling that was sufficient to induce adaptations comparable to those in the CHO periodized group. Some acute findings in highly trained endurance athletes support this idea by demonstrating similar increases in markers of mitochondrial biogenesis during recovery from glycogen depleting sessions, irrespective of the CHO intake post-exercise [30, 56]. Another explanation could be that the training interventions per se did fully exploit the adaptive response in most of the included studies, thus averting further improvements by CHO periodization (Table 1). In support hereof, significant improvements in endurance performance of 5–19% were observed in seven of the nine control groups training with high CHO availability [6, 8, 40–42, 45, 46] (Table 1). Performance improvements of such magnitudes could likely result from the use of intensified training programs, which was the case in at least three of the studies [6, 8, 45]. During periods containing this amount of high intensity training (e.g., 24x5min HIIT per week) it may be particularly difficult to achieve an additive training effect of CHO periodization. However, due to different periodization strategies, these intense periods are often conducted during certain parts of the season and surrounded by less intense periods where athletes are likely more stable in terms of performance and perhaps more responsive to “train-low” interventions [57, 58].

### Relevance of CHO periodization in highly trained endurance athletes

Elite coaches balance multiple components to optimize the overall physiological and mental stress among athletes (e.g., volume, intensity, distribution and recovery). Since superimposition of periodized CHO restriction onto routine training will likely affect the priority of other important parts of training, the potential benefits hereof must be carefully considered. In particular, the comprehensive strategies presented in the literature may be challenging to implement routinely among endurance athletes training 20–30 h each week (e.g., cyclists and triathletes). In this regard, it is worth noticing that the four studies using designs that reflect the actual training of elite endurance athletes have all shown that the effects of “real-life” training on performance were not augmented by CHO periodization [6, 8, 42, 45]. Also, many endurance athletes may already (in their habitual training) be exposed to prolonged exercise bouts with low glycogen levels towards the end or achieve the “train-low” effects during periods with frequent training bouts. Thus, endurance athletes are presumably already somewhat adapted to training with low CHO availability, and the potential for further effects from periodic CHO restriction may be limited, especially for those undertaking very long training bouts with low or no CHO intake. Such unconcious “exposure” to low CHO availability among endurance athletes can be caused by the difficulty of balancing energy intake and expenditure during periods with frequent training sessions and high training loads. While this may lead to an enhanced training response in some cases, low energy availability during prolonged periods may cause negative health-effects that will eventually compromise performance in other cases (e.g., endocrine perturbations and impaired bone health) [59]. This emphasizes the necessity of paying attention to the overall energy balance when introducing deliberate CHO restriction in endurance athletes with high training loads. In contrast, athletes with modest total training volumes and especially those consuming large amounts of CHO before and during training and recovery may be more likley to benefit from CHO periodization.

Based on the current literature, the use of CHO periodization is not clearly beneficial in elite endurance athletes. However, since both the intervention periods (i.e., one to four weeks) and performance tests (i.e., ≤ 2 h) used in the existing studies have been relatively short, it is too early to draw definitive conclusions. Except from a few studies [6, 8, 45], the training volume has also been lower than normal for elite endurance athletes in similar disciplines and the training intensities have often been clamped, which is deviating from the nature of “real-life” training in elite endurance athletes. In this regard and considering the relatively small potential effect that can be expected on performance in highly trained athletes, studies that superimpose tolerable CHO periodization approaches onto months of routine training are warranted.

As discussed above, a deliberate implementation strategy is important in order to avoid the potential negative consequences of training with low CHO availability. Thus, compromising training intensity by CHO restriction could be detrimental in endurance sports that are characterized by decisive periods of high exercise intensities (e.g., marathon running, road cycling and triathlon) and emphasizes the importance of prioritizing training quality in order to acquire training adaptations that promote the ability to perform such exercise [60–62]. Accordingly, the implementation of CHO periodization should be considered on both the macro-cycle level and micro-cycle level. As mentioned above, the relatively performance stable periods with low amounts of high intensity training may be preferable in terms of incorporating CHO periodization, and moreover, the risk of compromising training intensity is reduced during these periods. During training phases including larger amounts of high-intensity exercise CHO restriction must, on the other hand, be carefully incorporated to ensure enough resources to perform high-intensity training as well as appropriate recovery from this. In this regard, and based on the significant improvements observed in the control groups of the studies included in this review, it is likely that the conduction of traditional HIT sessions in a CHO-fed state elicits a sufficient near-maximal or maximal acute training response both on the muscle cell level and the cardiovascular level. This further supports the idea of incorporating “train-low” in training at lower intensities and future studies should examine whether CHO periodizing strategies proves beneficial during prolonged less intense training periods.

Finally, low CHO availability may reduce the training intensity during not only high-intensity exercise, but also moderate intensity exercise, which may compromise important training adaptations. Strategies that can partly or fully help to maintain the training intensity during “train-low” interventions are therefore warranted. For instance, it would be interesting to know if and to what extent CHO provision during training in a glycogen depleted state can rescue the training intensity (e.g., by reducing central fatigue) and how this affects the myocellular signalling in highly trained athletes.

## Conclusions

The potential physiological benefits associated with “train-low” has in recent years achieved increased scientific attention and with promising results from acute exercise studies. Accordingly, periodized CHO restriction has become common practice for some endurance athletes aiming to utilize this approach to enhance metabolic adaptations and endurance performance. However, based on the present meta-analysis it may be concluded that the evidence to support a performance enhancing effect of CHO periodization in well-trained endurance athletes is weak and “train-low” is not per se associated with enhanced endurance.

## Data Availability

Not applicable.

## Abbreviations

AT: Moderate-to-high intensity aerobic training
β-HAD: β-hydroxyacyl-CoA dehydrogenase
CHO: Carbohydrate
CS: Citrate synthase
HIIT: High intensity interval training
LCHF: Low carb high fat
LIT: Low intensity training
MAP: Maximal aerobic power
PEDro: The Physiotherapy Evidence Database
PO: Power output
RPE: Rate of perceived exertion
SMD: Standard mean difference
TT: Time trial
VO_2_max: Maximal oxygen consumption
